# Turning the corner on therapeutic cancer vaccines

**DOI:** 10.1038/s41541-019-0103-y

**Published:** 2019-02-08

**Authors:** Robert E. Hollingsworth, Kathrin Jansen

**Affiliations:** 1 Vaccines Research and Development, Pfizer, La Jolla, CA 92130 USA; 2 Vaccines Research and Development, Pfizer, Pearl River, NY 10965 USA

## Abstract

Recent advances in several areas are rekindling interest and enabling progress in the development of therapeutic cancer vaccines. These advances have been made in target selection, vaccine technology, and methods for reversing the immunosuppressive mechanisms exploited by cancers. Studies testing different tumor antigens have revealed target properties that yield high tumor versus normal cell specificity and adequate immunogenicity to affect clinical efficacy. A few tumor-associated antigens, normal host proteins that are abnormally expressed in cancer cells, have been demonstrated to serve as good targets for immunotherapies, although many do not possess the needed specificity or immunogenicity. Neoantigens, which arise from mutated proteins in cancer cells, are truly cancer-specific and can be highly immunogenic, though the vast majority are unique to each patient’s cancer and thus require development of personalized therapies. Lessons from previous cancer vaccine expeditions are teaching us the type and magnitude of immune responses needed, as well as vaccine technologies that can achieve these responses. For example, we are learning which vaccine approaches elicit the potent, balanced, and durable CD4 plus CD8 T cell expansion necessary for clinical efficacy. Exploration of interactions between the immune system and cancer has elucidated the adaptations that enable cancer cells to suppress and evade immune attack. This has led to breakthroughs in the development of new drugs, and, subsequently, to opportunities to combine these with cancer vaccines and dramatically increase patient responses. Here we review this recent progress, highlighting key steps that are bringing the promise of therapeutic cancer vaccines within reach.

## Introduction

In terms of lives saved, vaccines have been the greatest triumphs of medicine. Since their first use by Edward Jenner and his contemporaries, vaccines have been developed to prevent numerous infectious diseases by training the immune system to rapidly and specifically destroy the offending pathogen thus preventing disease. The application of vaccines to cancer is an obvious extension of their utility, but attempts to achieve this have been a frustrating journey. An exception is the generation of prophylactic vaccines against hepatitis B virus (HBV) and human papillomavirus (HPV), which are causes of liver and cervical cancer, respectively.^1,2^ These prophylactic vaccines have been successful because they circumvent three major challenges facing the development of therapeutic cancer vaccines: (1) low immunogenicity; (2) established disease burden; and (3) the immunosuppressive tumor microenvironment. Much of the work on therapeutic cancer vaccines has taken aim at tumor-associated antigens (TAAs), which are aberrantly expressed self-antigens. Since high-affinity T cells recognizing self-antigens are eliminated during development by our immune system’s central and peripheral tolerance mechanisms, TAA-directed cancer vaccines face the challenge of activating any remaining, low affinity T cells. To work in the therapeutic setting, vaccine-stimulated immune responses must be able to kill millions or even billions of cancer cells. In addition, research over the last decade has revealed many potent immunosuppressive mechanisms that evolve during the course of cancer progression. In many cases, these mechanisms rely on abnormal activation of suppressors that under normal conditions are involved in dampening a natural immune response once a pathogen has been cleared or a wound has healed. Furthermore, the immune system in many cancer patients has been severely debilitated due to aging, the side effects of cancer therapies, or immune cell exhaustion.^3–6^

Our rapidly increasing understanding of the biology of these obstacles has led to new approaches that are enabling researchers to turn the corner toward development of effective therapeutic cancer vaccines. Much of this new knowledge emanates from studies aimed at dissecting the interactions of the immune system and cancer, including the elucidation of how cancers exploit T cell checkpoint mechanisms. The development of checkpoint inhibitors (CPIs), the first of which were anti-CTLA-4, anti-PD-1, and anti-PD-L1 antibodies, represent a remarkable breakthrough in cancer medicine.^7^ Even so, these therapies are effective in only subsets of patients, and many patients who initially respond eventually relapse.^8,9^ Additional therapies are needed that can either elicit responses in patients who do not benefit from CPIs, or who do not benefit enough. Recent efforts focused on improving therapeutic cancer vaccine technology have been promising. In addition, intensive investigation into effective cancer vaccine targets has helped improve antigen selection, including more immunogenic and tumor-associated self-antigens, as well as neoantigens that harbor tumor-specific mutations. Combinations between CPIs and cancer vaccines are being tested as well. These efforts have brought about some encouraging clinical responses in patients. This review will summarize recent work and advances in target and antigen selection, cancer vaccine technologies, and combinations that may counteract the immunosuppressive tactics employed by tumors.

## Cancer vaccine antigens

**Fig. 1:**
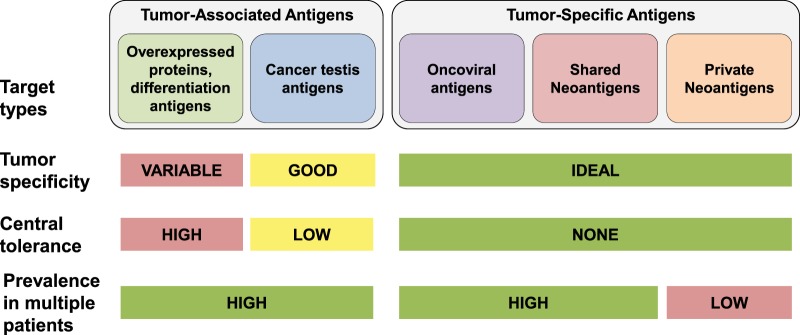
Therapeutic cancer vaccine target types. Targets for tumor vaccines fall into two general classes: tumor-associated antigens (TAAs) and tumor-specific antigens (TSAs). TAAs are self-antigens that are either preferentially or abnormally expressed in tumor cells, but may be expressed at some level in normal cells as well. As self-antigens, T cells that bind with high affinity to TAAs are typically deleted from the immune repertoire by central and peripheral tolerance mechanisms, and thus a cancer vaccine using these antigens must be potent enough to “break tolerance.” TSAs, comprised of antigens expressed by oncoviruses and neoantigens encoded by cancer mutations, are truly tumor-specific and as such high-affinity T cells may be present and strongly activated by these antigens. Although individual oncoviral antigens are expressed in specific tumor types (e.g., the HPV E6 and E7 antigens in cervical cancer), this occurs in many patients. Similarly, neoantigens encoded by oncogenic driver mutations may be prevalent across patients and tumor types, and hence are referred to as shared neoantigens. The majority of neoantigens are unique to individual patients’ tumors (private neoantigens), and thus require generation of a personalized therapy

The choice of antigen is the single most important component of cancer vaccine design. Ideally, the antigen should be expressed specifically by cancer cells (and not in normal cells), present on all cancer cells, necessary for cancer cell survival (such that the cancer cannot escape immune attack by downregulating the antigen), and highly immunogenic. Few if any antigens meet all of these criteria, yet there are several classes of antigens that have been employed in cancer vaccines (Fig. 1).

### Tumor-associated antigens

To date, most cancer vaccines have targeted TAAs, which are self-proteins that are abnormally expressed by cancer cells. TAAs include: cancer/germline antigens (also known as cancer/testis or CT antigens), which are normally expressed only in immune privileged germline cells (e.g., MAGE-A1, MAGE-A3, and NY-ESO-1);^10–14^ cell lineage differentiation antigens, which are normally not expressed in adult tissue (e.g., tyrosinase, gp100, MART-1, prostate-specific antigen (PSA), and prostatic acid phosphatase (PAP));^15–19^ and antigens that are overexpressed in cancer cells (e.g., hTERT, HER2, mesothelin, and MUC-1).^20–23^ Several hurdles are associated with developing vaccines against TAAs. First, as self-antigens, B cells and T cells that strongly recognize these antigens may have been removed from the immune repertoire by central and peripheral tolerance. Thus, a cancer vaccine must “break tolerance” by stimulating the low affinity or rare TAA-reactive T cells that remain.^24^ Strong adjuvants, co-stimulators, and repeated vaccination have been used to amplify the activation and expansion of self-antigen-reactive T cells,^25^ and this is particularly important for low-affinity T cells. Even with such enhancements, for many TAA-directed vaccine clinical trials the immune response, while detectable, does not appear to be strong enough to achieve significant efficacy. Often, such vaccines stimulate activation and proliferation of antigen-specific CD8 T cells to a level of <1% of the total circulating CD8 T cells, as compared to effective antiviral vaccines, which typically yield >5% antigen-specific CD8 T cells.^26^ For example, the YF-Vax yellow fever and the Dryvax smallpox vaccines stimulate expansion of activated antiviral CD8 T cells to 12.5 and 40% of total peripheral CD8 T cells, respectively,^26^ whereas PROSTVAC-VF, a metastatic prostate cancer vaccine targeting PSA induced antigen-specific T cell expansion to only about 0.03% of the total CD8 T cell population and was stopped in phase III due to futility.^27,28^ Although peripheral antigen-specific T cell counts may be useful, the number and quality of tumor-infiltrating T cells (TILs) are the more relevant measure, and patient TIL analysis is becoming more commonplace in cancer vaccine development. In either case, however, the specific T cell numbers needed for efficacy are currently unknown and undoubtedly varies by antigen, T cell receptor affinity, and tumor type. The good efficacy of antiviral vaccines as compared to failed TAA vaccines implies that the combination of T cell number, activation state, and antigen affinity must surpass a threshold to achieve clinical benefit, and simply detecting peripheral antigen-specific T cells is insufficient to predict efficacy.

A second challenge for targeting TAAs is that even if they are overexpressed on tumor cells, normal cell expression may lead to collateral damage. Although cancer vaccines have had acceptable tolerability so far, in many cases the vaccine was not efficacious and thus may have lacked potency. However, on-target off-tumor toxicity has been observed in clinical studies testing other therapies targeting TAAs. For instance, a chimeric antigen receptor-engineered T cell therapy (CAR-T) targeting the colorectal carcinoembryonic antigen (CEA) caused severe colitis in a high percentage of patients as this antigen is also expressed in normal intestinal tissue.^29^ As therapeutic cancer vaccines become more potent, close attention to normal cell toxicity will be important.

### Oncogenic viral antigens

Approximately 10% of human cancers worldwide are caused by viral infection, with most of these occurring in the developing world.^30^ As foreign antigens they can be highly immunogenic, and in some cases they are molecular drivers of oncogenesis. Vaccines comprised of HBV surface antigens have been shown to be highly effective in preventing infection and the associated disease sequelae, including hepatocellular carcinoma (HCC). Chronic HBV infection is a major contributor to HCC, and is implicated in up to 80% of adult HCC in regions where HBV is endemic.^31^ A national HBV immunization program launched in Taiwan in 1984 has resulted in a significant reduction in HCC.^32^ Vaccines comprising HPV-like particles also have provided remarkable protection against HPV infection and pre-cancerous lesions.^33–37^ Oncogenic HPV subtypes, especially HPV16 and HPV18, are a major cause of cervical cancer and also contribute to several other cancers. These prophylactic antiviral vaccines work by evoking production of potent neutralizing antibodies that prevent viral entry into host cells, and consequently prevent virus-mediated neoplasia. However, these prophylactic vaccines have not been effective in treating established cancer, most likely because humoral immunity cannot efficiently eradicate large numbers of virus-infected cancer cells, which instead requires cell-mediated immune responses. Consequently, distinct HPV vaccines targeting T cell epitopes of the viral E6 and E7 oncoproteins are being tested in clinical trials. These oncoproteins are expressed within infected cells and then processed and presented to stimulate cytotoxic T cells. Several different E6 and E7 vaccines are being tested in patients with cervical intraepithelial neoplasia (CIN), cervical cancer, and head and neck cancer. Early clinical results in CIN, an indolent form of the disease, have demonstrated tolerability, strong antigen-specific T cell responses, and encouraging signs of efficacy.^38–40^

### Neoantigens

Neoantigens that arise from cancer-specific mutations represent another class of attractive antigens for therapeutic cancer vaccines. Like viral oncoproteins, neoantigens are specific to tumor cells and are recognized as foreign by the immune system, and consequently reactive immune cells have not been eliminated by tolerance mechanisms. Some of the earliest evidence for neoantigens originates from studies of tumor rejection antigens in chemically and ultraviolet-induced cancers in mice.^41,42^ Numerous recent studies have shown that response to immune-mediated therapies, such as those targeting the CTLA-4 and PD-1 T cell checkpoint pathways, often correlates with high tumor mutation load^43–49^ as well as higher numbers of predicted neoantigens.^50–55^ Several groups have also shown that T cells in patients that respond to either CPIs, adoptive cell transfer of TILs, or dendritic cell (DC)-based cancer vaccines target neoantigens.^44,49,50,56–61^ Consequently, there is significant interest in developing and testing cancer vaccines targeting neoantigens. Several hotspot mutations commonly occurring in multiple cancer patients have been shown to encode immunogenic neoantigens, and adoptive cell therapy and therapeutic vaccines to a few of these have been found to elicit immunogenic and clinical responses.^62–64^ However, like cancer mutations, the majority of neoantigens are unique to each patient, and their numbers vary depending on tumor type. Generation of a cancer vaccine against a patient’s individual neoantigens thus requires a personalized approach: the patient’s tumor genome is sequenced, mutations are identified, neoantigens are predicted via computerized algorithms (and possibly confirmed experimentally to be expressed and bind major histocompatibility complex (MHC) proteins), and then a vaccine expressing the predicted neoantigens is constructed and delivered to the patient. Proof-of-concept studies in mice have tested the feasibility and efficacy of this approach.^65–67^ For example, Kreiter et al. found that vaccination with synthetic long peptide (SLP) neoantigens conferred potent antitumor activity in three different mouse models.^60^ In another murine study by Martin et al. vaccination with neoantigen-bearing SLPs induced robust T cell responses, but failed to prolong survival.^67^ Both studies made the unexpected observation that the majority of neoantigens stimulated CD4 rather than CD8 T cell responses. Two recent phase I clinical studies confirmed the potential of personalized neoantigen vaccines in melanoma patients.^68,69^ Ott et al. vaccinated six patients with SLPs containing up to 20 neoantigens that were specific to each patient’s tumor.^68^ Similarly, Sahin et al. tested RNA vaccines with up to 10 neoantigens per patient.^69^ In both cases, immune responses were detected in all patients, with activation of expansion of both CD4 and CD8 T cells reactive to multiple neoantigens. Clinical responses ranging from no recurrence in patients whose tumors had been resected to reduction in metastasis were observed in two-thirds of the patients, and several of the patients who relapsed then responded well to anti-PD-1 therapy. These early results are very encouraging and compel additional, larger trials and further development to optimize and reduce the cost and complexity of personalized neoantigen vaccines. In addition, common oncogenic driver mutations may give rise to neoantigens that are shared by multiple patients, and thus “off-the-shelf” vaccines employing these shared neoantigens are worth exploring.

## Vaccine vectors

**Fig. 2:**
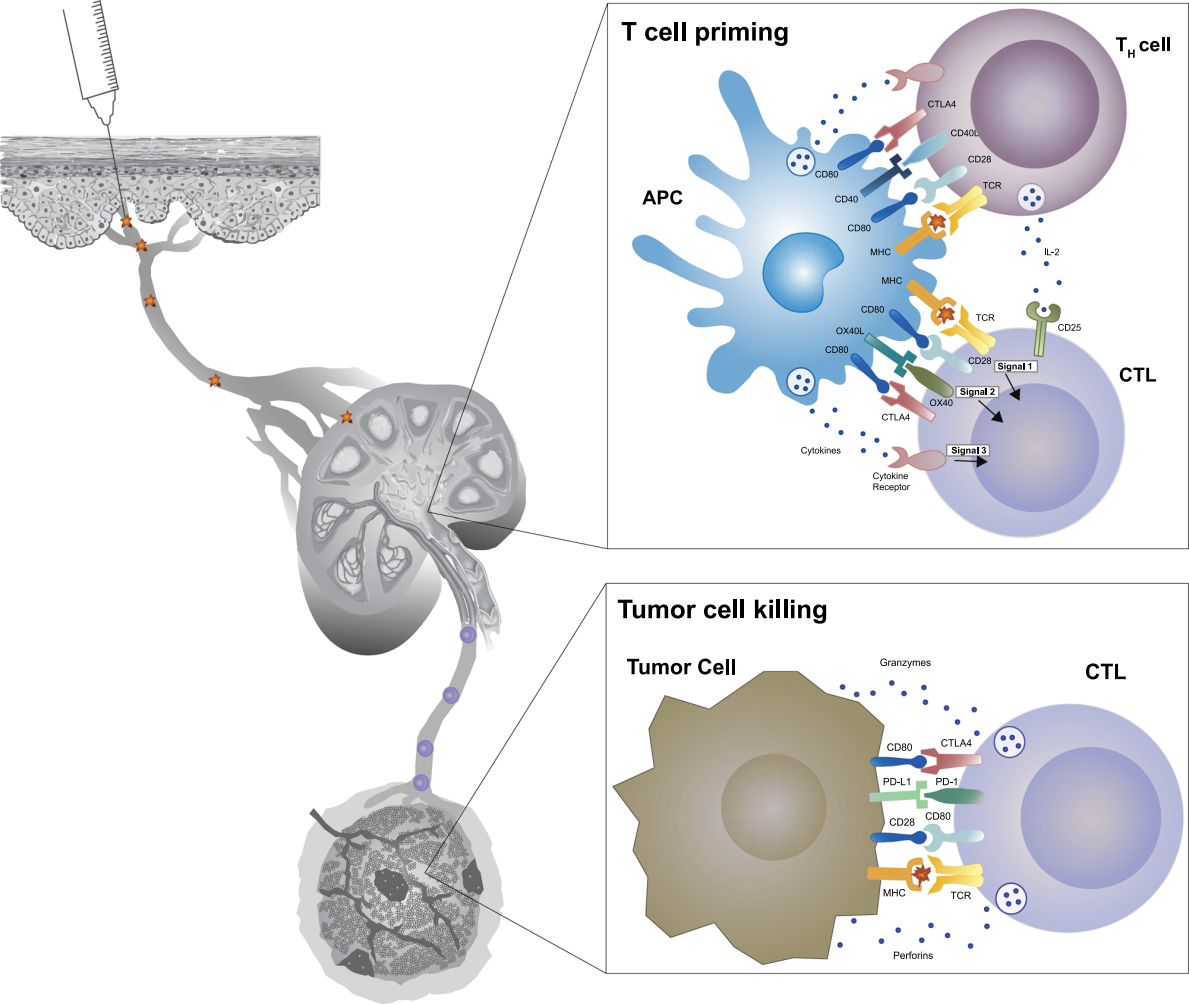
Mechanism of T cell activation and cancer cell killing. Activation of cytotoxic T cells (CTLs) depends on three signals: T cell receptor (TCR) engagement (signal 1), co-stimulation (signal 2), and an inflammatory stimulus (signal 3) via cytokines. T cell priming is initiated in tumor draining lymph nodes by specific binding of a TCR to its cognate peptide-major histocompatibility (MHC) complex displayed on an antigen-presenting cell (APC), particularly dendritic cells (DCs). This triggers a signaling cascade from the TCR complex that ultimately can regulate nuclear gene expression programs that transforms the T cell from a resting state to a state of activation and proliferation. Signal 1 alone, however, is insufficient for full activation, and the T cell must receive co-stimulation from the APC. Important co-stimulatory signals events include binding of the CD80 and CD86 ligands on APCs to the T cell CD28 receptor, and the binding of the OX40 and 4-1BB ligands to their receptors. TCR activation in the absence of co-stimulation can lead to T-cell anergy. In addition, co-stimulator ligands are depressed in tumors, and this can be overcome both by adjuvants that activate pattern recognition receptors on APCs, which upregulate expression of co-stimulatory ligands, and by antibodies that agonize the co-stimulatory receptors on T cells. Tumor cells also overexpress co-inhibitors, including CTLA-4 and PD-1, which normally function as T cell checkpoints to deactivate T cell activation after an infection is cleared. Antagonist antibodies have been developed to overcome this suppressive mechanism, and have demonstrated good clinical efficacy in some cancer types. Cytokines, including type I interferons and interleukin (IL)-12, provide the third necessary activation signal, and support the stimulation the expansion and differentiation of CD8 T cells into effector and memory CTLs. In addition, CD4 T_H_1 helper T cells can significantly amplify and sustain CTLs, primarily by supplying IL-2, whereas various other cells, including CD4 T_reg_ cells, myeloid-derived suppressor cells (MDSCs), and M2-type tumor-associated macrophages (TAMs) can significantly dampen CTL activation and function. T cells must also migrate to and infiltrate the tumor, and tumors employ numerous countermeasures to block this. These include physical barriers created by abnormal vasculature and stromal cell build up, as well as disruption of chemokines that guide T cells to the tumor

Several vaccine constructions have been tested for anticancer therapy, and lessons from these have pointed the way to substantial improvements. In each case, these vaccines are designed to present tumor-associated peptides complexed with MHC molecules to cognate receptors on B or T lymphocytes and stimulate their activation, maturation, and proliferation. Since tumor antigens are often derived from intracellular proteins, T cells are the most productive component of antitumor immune responses. Unfortunately, some of the early work in therapeutic cancer vaccine development followed the paths used for prophylactic vaccines for microbial infections, which work primarily through activation of B cell responses. Recent advances in therapeutic cancer vaccine technology have been bolstered by improved understanding of T cell activation and function (Fig. 2). In general, three types of vaccine platforms are being developed for cancer therapy: cellular vaccines, virus vector vaccines, and molecular vaccines comprised of either peptides, DNA, or RNA. All of these platforms have advantages and disadvantages, and are still being developed. Several excellent reviews have described each platform in detail,^70–74^ and here we will summarize recent developments and challenges.

### Cellular vaccines

Vaccines using either killed cancer cells or autologous antigen-presenting cells (APCs) loaded with cancer antigens have been developed and yielded some efficacy in patients.^75^ Both autologous patient-derived tumor cells and allogeneic cells derived from tumor cells lines, which are then irradiated to prevent further cell division, have been tested in preclinical and clinical experiments. An advantage of this approach is that target antigens do not have to be prospectively identified. The GVAX vaccines are composed of whole tumor cells genetically modified to secrete the immune stimulatory cytokine, granulocyte-macrophage colony-stimulating factor (GM-CSF), a potent immunostimulatory cytokine that promotes antigen presentation, activation, and survival of DCs.^76^ Although such vaccines have worked well in murine tumor models, inducing immune responses and tumor regression,^77–80^ several clinical trials testing GVAX vaccines in prostate cancer, melanoma, pancreatic cancer, and lung cancer have demonstrated only limited efficacy despite stimulating immune responses in patients.^81–84^ For example, two phase III studies testing an allogeneic prostate cancer cell line GVAX were terminated early due to lack of therapeutic effect.^85,86^

DC vaccines, in which patient-derived, autologous DCs are either loaded with peptide antigen or transfected with antigen genes, have also been studied extensively.^87^ The first US Food and Drug Administration-approved cancer vaccine, sipuleucel-T (Provenge), is in use for metastatic castration-resistant prostate cancer (mCRPC). This vaccine is generated by enriching DCs derived from each patient by leukapheresis and activation ex vivo with a chimeric protein, GM-CSF fused to the antigen PAP. A pivotal phase III clinical trial (IMPACT) randomized 512 mCRPC patients 2:1 to receive sipuleucel-T or placebo.^88^ This study demonstrated a small but significant increase in median overall survival (OS) of 25.8 versus 21.7 months for placebo. The toxicity profile was good, with transient flu-like symptoms and fever being the most common side effects. However, the complexity and price of producing sipuleucel-T has been an obstacle to its widespread use. Nonetheless, sipuleucel-T serves to demonstrate that autologous DC vaccines can work. Several other DC vaccines are being developed and tested. For example, phase I and II trials using autologous DCs pulsed with melanoma antigen MART-1_27-35_ peptide or transduced with an adenovirus encoding full-length MART-1 yielded promising results.^89,90^ In these studies, patients with the best clinical outcomes had evidence of epitope spreading to other melanoma-associated antigens.

Other cellular vaccines employ microorganisms (bacteria or yeast) to stimulate an immune response or to deliver tumor antigens. Coley was the first to use a heat-inactivated bacterial mixture that produced significant responses in cancer patients,^91^ probably driven by activating cytokine production, which then stimulated antitumor immune responses. Bacillus Calmette-Guérin, a live attenuated strain of *Mycobacterium bovis*, has been in use for more than 40 years to treat bladder carcinoma in situ, and although its full mechanism remains unclear, it functions in part to stimulate an antitumor immune response.^92^ Other species of bacteria such as *Lactococcus*, *Listeria*, *Salmonella*, *and Shigella* have been used as anti-infective and anticancer vaccine vectors as well.^93–95^ For example, attenuated strains of *Listeria monocytogenes*, which infect and are internalized by various cell types including APCs, can deliver DNA- or RNA-encoded tumor antigens and induce potent antitumor immunity and efficacy in preclinical models.^96,97^ Recent clinical trials of attenuated *Listeria*-based cancer vaccines have established acceptable safety and immune activation,^97^ however, clear and reproducible efficacy remains to be seen. For example, encouraging phase II efficacy data testing a heterologous cellular vaccine comprised of GVAX and *Listeria* expressing mesothelin (CRS-207) in patients with advanced pancreatic cancer^98^ were not be reproduced in a larger phase IIb study,^99^ and the development of the CRS-207 has been abandoned.^100^

### Peptide vaccines

Many peptide vaccine clinical trials have been conducted with demonstration of immune responses, yet significant clinical benefit has been elusive. Often only single antigen-based short peptides were used, which may not be able to overcome antigen heterogeneity or loss of antigen expression within the tumor or stimulate robust immune responses.^101,102^ Short peptides (<15 amino acids) do not require processing by APCs and can bind effectively to MHC class I molecules on the surface of many nucleated cells. However, binding to and presentation by cells other than professional APCs that do not provide proper co-stimulation leads to a tolerogenic signal and T cell dysfunction.^103–106^ In addition, short peptides do not activate CD4 helper T cells, which are necessary for full activation of cytotoxic T lymphocytes (CTLs). Efforts to improve the potency and quality of peptide vaccines have included constructs with amphiphilic peptides, peptide fusions to Toll-like receptor (TLR) agonists, addition of powerful inflammatory adjuvants, and combinations with other immune modulators.^107–112^ For example, the TLR3 ligands Poly-IC and Poly-ICLC have proven to be successful adjuvants in experimental models when combined with peptide vaccines.^108,113,114^ In comparison to short peptides, the use of multivalent synthetic long peptides (SLPs), containing both MHC class I and class II epitopes, can elicit a balanced induction of both CD8 and CD4 T cells.^115^ Moreover, SLPs are preferentially taken up and processed by DCs, rather than by other APCs, which lack co-stimulatory molecules, leading to more productive T cell priming and induction of memory T cell responses, probably due activation of CD4 helper T cells.^103,116,117^

### Viral vector vaccines

Several viruses have been exploited as cancer vaccine platforms.^118^ An advantage for virus-based vaccines is that the immune system has evolved to efficiently respond to viruses, with both innate and adaptive mechanisms working in concert to effect a strong and durable response. Viral pathogen-associated molecular patterns trigger pattern recognition receptors (PRRs) to boost activation of APCs. The most commonly used viral vaccine vectors have been derived from poxviruses, adenoviruses, and alphaviruses, and replication-defective or attenuated versions are preferred for safety. A disadvantage of viral vectors is that the antiviral immune response neutralizes the vector, thus limiting repeat vaccination. To address this, a heterologous prime-boost strategy is often used where a tumor antigen is delivered with one virus vector first, followed by a boost with the same tumor antigen delivered by a different viral vector or vector type (e.g., DNA plasmid). PROSTVAC-VF/Tricom is a good example of this approach. This vaccine regimen utilizes a vaccinia virus encoding PSA antigen for priming, followed by six subsequent booster doses of a fowlpox virus encoding PSA.^119^ In a phase II randomized trial in 125 men with mCRPC, PROSTVAC in combination with GM-CSF yielded a 10-month longer OS compared to the empty vector control group (26.2 versus 16.3 months).^120^ Unfortunately, these promising findings were not replicated in a large phase III trial, where the regimen was deemed unlikely to improve OS compared to the control, and the trial was stopped.^28^ Although PROSTVAC activates PSA-specific T cells,^121^ this activation is either not sufficiently potent and/or the vaccine alone is incapable of overcoming the tumor immunosuppressive microenvironment to achieve significant efficacy in mCRPC patients. Consequently, PROSTVAC is now being tested in combination with several CPIs [NCT02933255 and NCT02506114].

To increase cancer vaccine potency and overcome the tumor immunosuppressive environment, we have developed VBIR, a vaccine-based immunotherapy regimen, which combines a heterologous prime-boost vaccine with CPIs (Cho et al., personal communication, and ref. ^122)^. A priming vaccination is accomplished with a replication-defective chimpanzee adenovirus (ChAd68 serotype), and use of a non-human-specific virus circumvents pre-existing immunity. Boosting is then conducted by delivery of a DNA plasmid encoding the same antigens by intramuscular electroporation. Local subcutaneous injections of tremelimumab, a CTLA-4 antagonist antibody, and RN888, a PD-1 antagonist antibody, are given concurrently with the vaccinations to enhance T cell priming and prolong activity. VBIR induces strong and durable expansion of polyfunctional (IFNγ+, TNFα+, and/or IL-2+) T cells in mice and monkeys, and produces tumor regressions in mouse cancer models. A VBIR expressing the antigens PSA, prostate-specific membrane antigen (PSMA), and prostate stem cell antigen (PSCA is undergoing phase I clinical testing currently [NCT02616185].

### DNA and RNA vaccines

Like peptide vaccines, DNA and RNA vaccines have the advantage of comparatively simple and inexpensive production. However, they can also trip nucleic acid sensors that activate DCs, including certain TLRs, STING, AIM2, and DAI pathways, and so their need for adjuvants is less critical. In addition, nucleic acid vaccines can be dosed repeatedly since they do not provoke strong anti-vector immunity. Naked DNA and RNA can be taken up by various APC types, including myocytes, monocytes, and DCs. The efficiency of cellular uptake of naked nucleic acids is low, so developing formulations and techniques to improve uptake (besides viral vectors as described above) has been a research focus. Delivery by nanoparticles, gene gun, microneedle arrays, and in situ electroporation has been found to dramatically improve transfection.^123^ For instance, electroporation of DNA vaccines can increase immunogenicity by 100- to 1000-fold over direct injection.^124^ This improved immunogenicity leads to stronger tumor killing in mouse models, and several electroporated DNA vaccines are now being studied in cancer patients, either as standalone vaccines or as part of a heterologous prime-boost approach. Among the DNA vaccines that have advanced the farthest in clinical testing, VGX-3100, expressing the HPV E6 and E7 antigens, is being tested in a phase III trial in high-grade squamous HPV-positive cervical cancer (NCT03185013). A previous phase IIb trial demonstrated the first significant efficacy of a therapeutic cervical cancer vaccine, with tumor regression seen in 48.2% of treated women with grade 2/3 CIN compared to 30.0% in the placebo group.^125^

RNA cancer vaccines may offer advantages over DNA vaccines.^126^ While RNA is more susceptible to degradation by ubiquitous RNases, this can be mitigated by chemical modifications and incorporation of modified nucleosides such as pseudouridine.^127,128^ Unlike DNA, RNA cannot integrate into the genome and therefore has no oncogenic potential. Moreover, RNA needs only to enter the cytoplasm, whereas DNA needs to enter the nucleus thus facing an additional barrier, the nuclear membrane. To date, the majority of RNA vaccines being tested in the clinic have employed mRNA, however, the use of RNA replicons is now being explored as well.^129^ Replicon RNA is self-replicating, and thus persists in the cell longer than mRNA and requires lower vaccination doses. To improve transfection efficiency and further avoid degradation, many groups have tested different delivery methods for RNA vaccines, including some of the methods described above for DNA delivery, but also condensation with protamine and encapsulation into liposomes or nanoparticles.^130–135^ A recent encouraging phase I trial in advanced melanoma patients evaluated mRNA expressing four different TAAs complexed in a liposomal formulation.^136^ Patients developed de novo T cell responses to the antigens and either regression of metastatic lesions or disease stabilization.

## Combinations with other therapies

Cancers arise despite the presence of immunosurveillance that routinely detects and eliminates abnormal cells, indicating the evolution of immunosuppressive and evasive mechanisms. Recent research has uncovered numerous such mechanisms, and most cases cancers employ several of them. This has sparked an explosion of work to develop anticancer immunomodulators. The development of CPIs, especially CTLA-4 and PD-1/PD-L1 antagonists, not only catalyzed progress in immuno-oncology, but now offers key ingredients for maximizing the efficacy of therapeutic cancer vaccines. Any therapy that induces more effective tumor-specific T cell responses should synergize with CPIs, with vaccination being a prime example. This could be especially effective for patients harboring particularly weak spontaneous T cell responses to their cancer. Multiple preclinical studies have shown synergy between therapeutic vaccination and CPIs,^137,138^ and several clinical studies are now evaluating this. A recent phase II trial testing the addition of the PD-1 antagonist, nivolumab, to a multi-antigen HPV E6 and E7 vaccine yielded a median OS nearly double that of CPIs alone in incurable HPV-positive oropharyngeal cancer patients.^139^ Besides CTLA-4 and PD-1/PD-L1 inhibitors, several other CPIs are being developed, including inhibitors of TIM-3 and LAG-3. In a phase I trial testing the combination of a LAG-3 antagonist antibody with a MART-1 peptide vaccine, five out of six melanoma patients had elevated MART-1-specific T cell responses versus one out of six patients treated with the vaccine alone.^140^ However, not all combinations between cancer vaccines and CPIs have improved responses. In the landmark phase III trial demonstrating an OS benefit to melanoma patients treated with ipilimumab (a CTLA-4 antagonist antibody), Hodi et al. found no added benefit when a gp100 peptide vaccine was added.^141^ This was likely due to the use of a short peptide vaccine, which we now know has low potency compared to other vaccine technologies.

Immunostimulators, including adjuvants, cytokines, and other agents, can also improve the efficacy of cancer vaccines. Indeed, delivery of an antigen without appropriate co-stimulators results in T cell ignorance, T cell anergy, or even T cell deletion.^106^ The immune system probably evolved to defend against microbial pathogens, and in order for a vaccine to mimic an invading microbe it must be recognized as both foreign and dangerous. Viral, bacterial, and nucleic acid vaccine technologies can provide both of these signals, but peptide vaccines do not provide the requisite danger signal. Consequently, much effort has been devoted to developing strong adjuvants that emulate pathogen- and damage-associated molecules recognized by PRRs, including the TLRs. A wide variety of adjuvants that can trigger PRRs have been used in preclinical cancer vaccines studies, and some have been tested in clinical trials.^106,142,143^ These adjuvants induce local inflammation and cytokine production, activation of APCs, and increased magnitude and function of T cell responses.

Potent T cell activation also requires engagement by costimulators on APCs and CD4 helper T cells (“signal 2”, with presentation of the peptide-MHC complex being “signal 1”), and stimulatory cytokines (“signal 3”) (Fig. 2). Costimulators include members of the tumor necrosis factor receptor family, such as OX40, 4-1BB, and CD40.^144^ Therapeutic activation of these costimulators may thus potentiate the activity of cancer vaccines, and experiments in murine cancer models support this notion. Murata et al. showed that the combination of a whole tumor cell vaccine with an OX40 agonist antibody effectively induced a durable antigen-specific CD8 T cell response despite established immune tolerance to the target antigen.^145^ Agonist OX40 antibodies also enhanced the CD4 and CD8 T cell response generated by a DC-based vaccine.^146^ Various triple combinations of cancer vaccines with CPIs and costimulator agonist antibodies are also being explored. Vaccination with an adenovirus-based vaccine delayed tumor growth in 30–40% of mice, while vaccination in combination with anti-CD40 and anti-CTLA-4 antibodies induced complete responses in all mice.^147^ In another study, mice treated with anti-CTLA-4 and an OX40 agonist antibody plus a DC vaccine targeting HER2 had significantly improved survival in a mammary carcinoma model.^148^

Cytokines are also being explored as therapeutic cancer vaccine combination partners. Using a similar gp100 peptide vaccine to that used in the Hodi et al. ipilimumab trial, Schwartzentruber and colleagues evaluated the vaccine in combination with high-dose interleukin-2 (IL-2) in patients with advanced melanoma.^149^ In this randomized phase III clinical trial, the combination showed a significant improvement in objective response rate and progression-free survival (PFS) compared to patients treated with IL-2 alone. The adverse effects were similar in both groups with most toxicities attributed to IL-2. Emerging preclinical results suggest that other cytokines, particularly IL-12 and IL-15, will combine effectively with cancer vaccines, either dosed separately or expressed within a cellular vaccine.^150,151^ However, clinical trial results testing combination with these other cytokines have not been reported.

Finally, combinations of cancer vaccines with chemo- and radiotherapies have been described in many recent reports. Because the standard of care for many cancers includes chemo- and radiotherapies, development of most cancer vaccines will involve patients that have either been treated or are concurrently being treated with these therapies, and thus understanding their interactions with cancer vaccines is crucial. Radiotherapy has been shown to induce immunogenic tumor cell stress and cell death, resulting in enhanced sensitivity to T cells and synergy with cancer vaccines in preclinical studies.^152,153^ A few early clinical trials testing radiotherapy plus cancer vaccine combinations have been initiated, but results are still emerging.^154^ Evidence accumulating over the last several years has also indicated that the efficacy of some conventional chemotherapy agents not only involves direct cytostatic/cytotoxic effects but also relies on modulation of the immune system.^155^ Combinations between CPIs and chemotherapies are now been vigorously explored and have begun to show promise; for example, in a recent clinical trial in non-small cell lung cancer patients, the addition of pembrolizumab to standard chemotherapy resulted in significantly longer OS than chemotherapy alone.^156^ Multiple studies in animal models have shown that chemotherapy can combine effectively with cancer vaccines as well,^157^ and this has motivated clinical testing. A study by Welters et al. explored the combination of an HPV16 SLP vaccine with standard carboplatin and paclitaxel chemotherapy in the HPV16 E6/E7-positive TC-1 mouse tumor model and in patients with advanced cervical cancer.^158^ Results showed that the chemotherapy reduced the numbers of immunosuppressive myeloid cells in tumors and in the blood, and this fostered vigorous vaccine-induced T cell responses. In another example, a phase IIb/III trial in advanced non-small cell lung carcinoma tested the combination of TG4010, a modified Ankara virus vaccine expressing MUC-1 and IL-2, and platinum-based chemotherapy.^159^ In this study the combination group had a longer median PFS and more confirmed responses compared to chemotherapy alone. Although these cases are encouraging, more work needs to be done to understand and optimize such combinations with cancer vaccines.

## Conclusions and perspectives

Cancer immunotherapy has experienced tremendous progress in the last decade, including dramatic expansion of our understanding of how cancer cells evade the immune system and the development of several new therapies that are benefitting cancer patients. In particular, antibodies that modulate the function of the CTLA-4 and PD-1 T cell checkpoints have produced durable tumor regressions in some patients, including some for whom other therapies were ineffective. Adoptive T cell therapies have also produced remarkable responses, in particular CAR-T therapy in acute lymphocytic leukemia and TIL therapy in melanoma. These breakthroughs have proven the feasibility and efficacy of cancer immunotherapies, and opened new paths to develop other new medicines. However, many patients do not respond to current immunotherapies, and most that do eventually relapse; in addition, many patients experience adverse effects with current therapies. Therapeutic cancer vaccines offer an attractive alternative immunotherapy because of their potential safety, specificity, and long-lasting response—perhaps even cures—due to stimulation of immune memory. Unfortunately, many previous attempts to develop effective therapeutic cancer vaccines yielded disappointing results. Lessons learned from these failed attempts are now allowing cancer vaccine research to turn the corner and begin to achieve some promising clinical results. The key lessons driving this progress emanate from three areas: the need for multiple, immunogenic antigens; the importance of highly potent vaccine vectors; and a growing understanding of how to quell tumor-mediated immunosuppression. New strategies are enabling the selection and construction of more immunogenic TAAs and the identification of tumor-specific neoantigens. Enhanced vaccine technologies, including viral vector prime-boost approaches, better co-stimulatory components, multi-antigen vaccines, and stimulation of both CD8 and CD4 T cells responses, are being tested. Additionally, combinations with checkpoint modulators and other new drugs that reverse immunosuppression are showing promise, although much work needs to be done to determine which combinations are most effective and the optimal dose scheduling for each component. In our opinion, the simultaneous application of improvements in all three areas will be required for cancer vaccines to be successful.

Other significant problems need to be solved in order to maximize success as well. One major deficiency is the current lack of validated biomarkers that predict vaccine efficacy and that can be used to guide optimization. Vaccine-induced TIL increase is an obvious possibility, but the quantity and quality of TILs required for clinical efficacy is still unknown and probably varies for different vaccines and cancer settings. A clear understanding of which subtypes of T cells are most important for an effective cancer vaccine, and how to more specifically stimulate these, is needed. For example, recent studies have suggested the importance of resident memory T cells for the efficacy of immunotherapies.^160,161^ Downregulation of MHC class I molecules, which are involved in antigen presentation, appears to be a common immuno-evasion mechanism in cancer,^162,163^ so finding new approaches to counteracting this will be important and combinations of cancer vaccines with agents that increase MHC expression in cancer cells should be explored.^164–166^ Some of our new approaches are creating new challenges as well. For example, the production of personalized vaccines bearing patient-specific neoantigens is a technically complex, time-consuming, and expensive task. Likewise, the testing and optimizing vaccine plus immunomodulator drug combinations in clinical trials will require considerable time and effort. The recent, albeit nascent, success seen in several clinical trials provides impetus to tackle these challenges and reason for optimism that therapeutic vaccines will soon become an important new addition to cancer immunotherapy.
